# Biomechanics of artificial pedicle fixation in a 3D-printed prosthesis after total en bloc spondylectomy: a finite element analysis

**DOI:** 10.1186/s13018-021-02354-0

**Published:** 2021-03-24

**Authors:** Xiaodong Wang, Hanpeng Xu, Ye Han, Jincheng Wu, Yang Song, Yuanyuan Jiang, Jianzhong Wang, Jun Miao

**Affiliations:** 1 Graduate School, Tianjin Medical University, Tianjin, China; 2 Department of Orthopaedics, Affiliated Hospital of Hebei University, Baoding, China; 3 Department of Orthopaedics, Tianjin Hospital, Tianjin, China

**Keywords:** 3D-printed prosthesis, TES, Spinal stability, Finite element analysis

## Abstract

**Background:**

This study compared the biomechanics of artificial pedicle fixation in spine reconstruction with a 3-dimensional (3D)-printed prosthesis after total en bloc spondylectomy (TES) by finite element analysis.

**Methods:**

A thoracolumbar (T10–L2) finite element model was developed and validated. Two models of T12 TES were established in combination with different fixation methods: Model A consisted of long-segment posterior fixation (T10/11, L1/2) + 3D-printed prosthesis; and Model B consisted of Model A + two artificial pedicle fixation screws. The models were evaluated with an applied of 7.5 N·m and axial force of 200 N. We recorded and analyzed the following: (1) stiffness of the two fixation systems, (2) hardware stress in the two fixation systems, and (3) stress on the endplate adjacent to the 3D-printed prosthesis.

**Results:**

The fixation strength of Model B was enhanced by the screws in the artificial pedicle, which was mainly manifested as an improvement in rotational stability. The stress transmission of the artificial pedicle fixation screws reduced the stress on the posterior rods and endplate adjacent to the 3D-printed prosthesis in all directions of motion, especially in rotation.

**Conclusions:**

After TES, the posterior long-segment fixation combined with the anterior 3D printed prosthesis could maintain postoperative spinal stability, but adding artificial pedicle fixation increased the stability of the fixation system and reduced the risk of prosthesis subsidence and instrumentation failure.

## Introduction

Total en bloc spondylectomy (TES) is an effective treatment for primary and metastatic malignant spinal tumors as it greatly reduces local recurrence and improves patients’ quality of life [1–3] and prolongs their survival [4, 5]. However, total resection of single or multiple vertebral bodies and surrounding ligaments leads to severe instability of the spinal segments. A solid vertebral body replacement (VBR) and long-segment posterior fixation are needed to achieve stable spinal reconstruction and preserve spinal function [5, 6]

There are currently many options for VBR, which mainly depends on the axial pressure of the vertebral body and tightening force of pedicle screws for stability. VBR subsidence is the main technical complication after TES, which can lead to rod breakage and fixation failure [7–9]. Colman carried out a TES model experiment and showed that application of a VBR with an artificial pedicle fixed to the posterior rod greatly enhanced the stability of the fixation system and prevented subsidence [10]. Artificial pedicle fixation not only prevented VBR displacement, but also transferred stress and distributed the load between internal fixations [10]. Simple artificial pedicle connections have been used to improve fixation stability [11, 12]. However, because of the limitations of off-the-shelf VBR designs, a firm connection is rarely achieved.

Three-dimensional (3D)-printed prostheses can be used to design additional anchor sites for the implant to increase the stability of the system including customized bionic artificial pedicles. Artificial pedicles can integrate the VBR and posterior rods. To date, there are several reports of good results achieved using 3D-printed prostheses with artificial pedicle fixation for spine reconstruction after TES [13–15]. However, there have been no studies investigating the biomechanical effects of artificial pedicles on internal fixation systems following TES. To address this point, in this study, we used a 3D finite element model of the thoracolumbar spine to simulate and analyze the biomechanics of artificial pedicle fixation in spine reconstruction after one-level TES.

## Materials and methods

### Normal finite element model

**Fig. 1:**
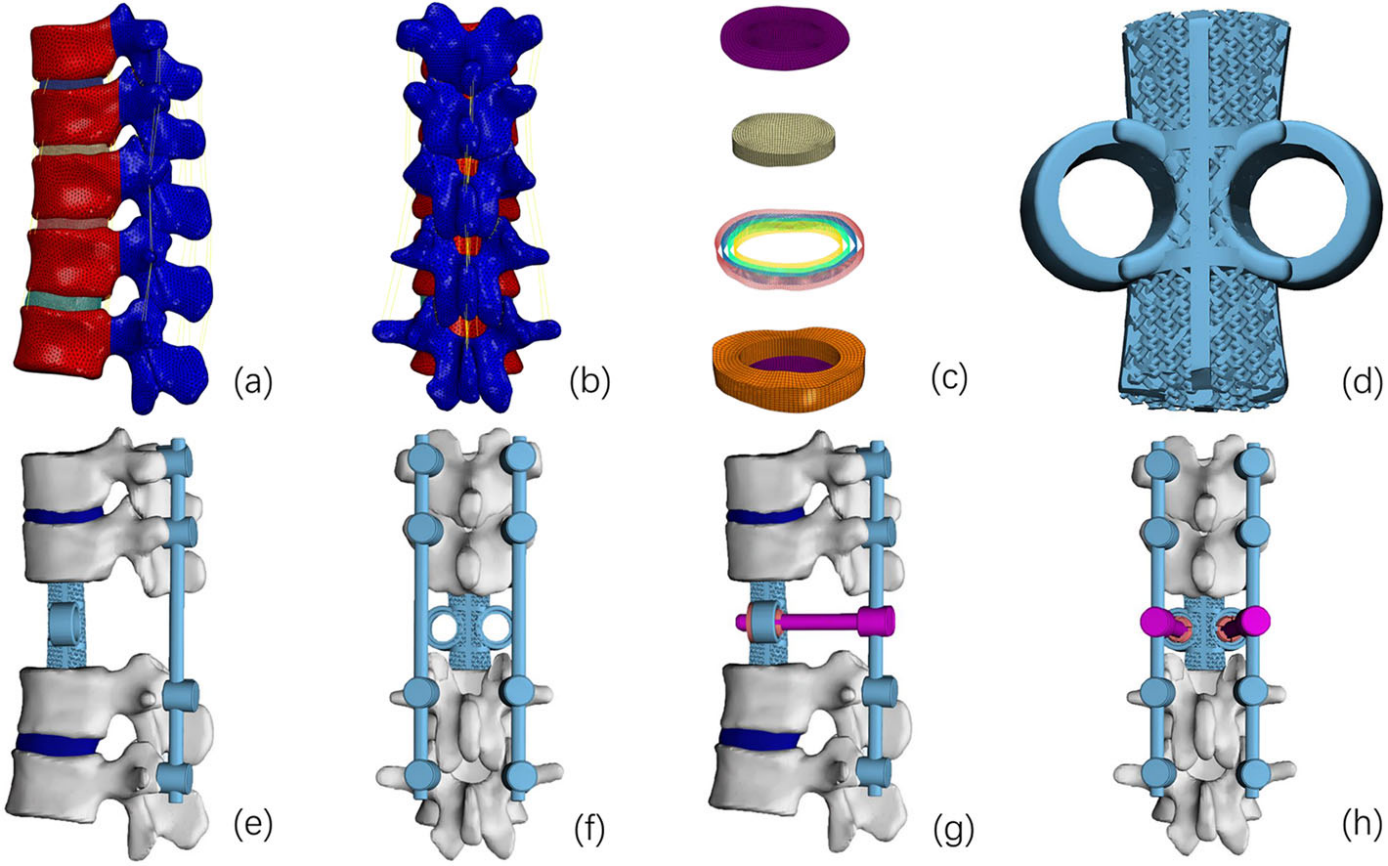
Different 3D models. **a**, **b** Intact model of T10–L2 including major ligaments. **c** Structure of the IVD. **d** Model of the 3D-printed prosthesis with artificial pedicle structure. **e**, **f** Model A: 3D-printed prosthesis with long-segment posterior fixation (T10/11 and L1/2). **g**, **h** Model B: Model A + two artificial pedicle fixation screws

We selected a healthy male volunteer to generate a normal finite element model. The image data in DICOM format of five vertebrae and four discs between T10 and L2 were obtained with a 64-slice spiral computed tomography scanner (Siemens, Erlangen, Germany) at 1-mm interlayer spacing. We imported the images into Mimics v20.0 (Materialise, Leuven, Belgium) to create 3D vertebral surface models of T10 to L2 in STL format. The posterior structure and intervertebral discs (IVDs) were constructed using 3-matic v12.0 (Materialise) [16–18]. The models were imported into Geomagic Studio v12.0 (Geomagic, Research Triangle Park, NC, USA) and processed by smoothing and surface and grid construction. The bone and ligament structures were meshed using Hypermesh 2017 (Altair Engineering, Troy, MI, USA). Abaqus 2019 (Simulia, Johnston, RI, USA) was used for material property definition, model assembly, loading, and finite element analysis. The intact T10–L2 finite element model is shown in Fig. 1. After mesh convergence, the mesh sizes of the vertebral body and IVD were 1.5 and 1 mm, respectively. The cortical bone, facet joint, and cartilage endplate were simulated with shell elements with thicknesses of 1, 0.2, and 0.5 mm, respectively [16, 17]. IVDs were divided into nucleus pulposus, annulus fibrosus, and endplates. The nucleus pulposus accounts for 30–40% of intervertebral volume. The annulus fibrosus is composed of the annulus fibrosus matrix and fibers that are divided into three–five layers at an angle of 30° [16, 18]. Seven ligaments (anterior longitudinal, posterior longitudinal, interspinous, supraspinous, intertransverse, and capsular ligaments and ligamentum flavum) were created for each segment [16]. Ligaments and fibers were simulated using T3D2 elements.

### Implants and fixation models

SolidWorks (Dassault Systemes, Paris, France) was used to draw pedicle screws (6.5 × 45 mm, 6.0 × 40 mm), rods (5.5 mm), and a 3D-printed prosthesis with an artificial pedicle structure (AK Medical, Beijing, China) (35 × 20 × 15 mm) according to the size of the 3D finite element model, which was validated using Geomagic Studio v12.0. The material properties used in the finite element model (Table 1) were based on previous reports [19, 20].

**Table 1:** Material properties for the thoracolumbar spine finite element model

Structure	Young’s modulus (MPa)	Poisson ratio	Cross-sectional area (mm^**2**^)
Vertebrae			
Cancellous bone	100	0.2	
Cortical bone	12,000	0.3	
Posterior elements	3500	0.25	
Disc			
Annulus	4.2	0.45	
Nucleus	0.2	0.49	
Facet	11	0.2	
Ligaments			
Anterior longitudinal ligament	7		63.7
Posterior longitudinal ligament	7		20
Ligamentum flavum	3		40
Intratransverse ligament	7		1.8
Capsular ligament	4		30
Interspinous ligament	6		40
Supraspinous ligament	6.6		30
Pedicle screw and rod fixation	110,000	0.3	
3D-printed prosthesis	675	0.3	

A T12 TES model was created using 3-matic v12.0 with the whole vertebra and adjacent IVD (T11/12, T12/L1 IVD) removed. Two surgical models were constructed (Fig. 1), each using a different combination of screws. In Model A, the 3D-printed prosthesis was fixed with long-segment posterior fixation (with two-level pedicle screw fixation above and below the VBR; T10/11 and L1/2) and in Model B, artificial pedicle screws connected the 3D-printed prosthesis in Model A (T10/T11, L1/2, and the 3D-printed prosthesis). The pedicle screws inserted into the vertebral body were 6.0 × 40 mm, and the pedicle screws inserted into the 3D-printed prosthesis were 6.5 × 45 mm.

### Boundary and loading conditions

Abaqus 2019 was used to set boundary and load conditions and simulate spinal movement. We assumed that the L2 vertebral body was fixed, and its substructure was set as a boundary with no displacement or rotation in any direction. Spinal motion in sagittal, coronal, and cross-sections was defined as flexion, extension, lateral bending, and rotation. We applied an axial load of 200 N and torque load of 7.5 N·m to the upper surface coupling point of T10 to simulate the flexion, extension, lateral bending, and rotation of the spine [21, 22].

### Assessment indices

Three indices were used to assess the mechanical properties of the structure: stiffness of the construct (T10–L2), von Mises stress of the internal fixed system, and von Mises stress on the endplate adjacent to the 3D-printed prosthesis (L1 superior endplate). We used these indices to evaluate the biomechanical effects of the artificial pedicle in the constructed models. Since only one subject was modeled, no statistical analysis was performed in this study.

## Results

### Validation of the intact model

**Fig. 2:**
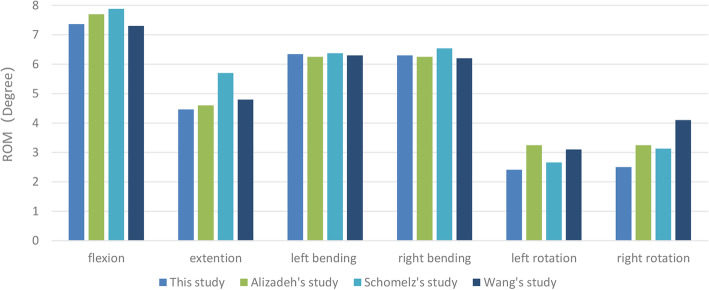
Comparison between ROM values from the T12–L2 thoracolumbar model in this study and previously reported values

Figure 2 shows the comparison between the range of motion (ROM) values for T12–L2 junctions obtained in this study and previously published data from biomechanical and finite element analysis experiments measuring flexion, extension, lateral bending, and axial rotation. The ROM for T12–L2 junctions predicted by the model were in agreement with experimental data from previous studies [23–25], thus validating the current thoracolumbar model.

### Stiffness of the thoracolumbar junction

**Fig. 3:**
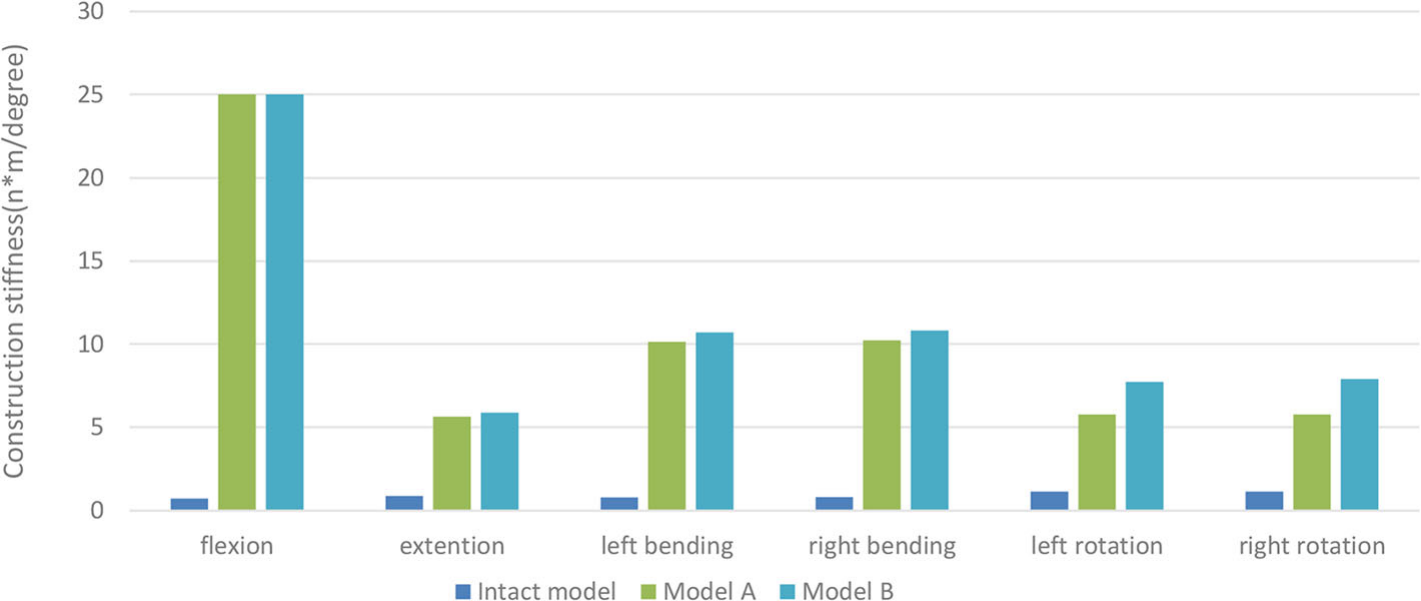
Stiffness of the intact thoracolumbar model (T10–L2) and two fixation models

The two fixation models showed a much higher stiffness than the intact model (Fig. 3). Because of the buttress provided by the VBR, the two fixation models showed a maximal and equal stiffness of 25.0 N·m/° in flexion. In other types of motion (especially rotation), the stiffness of Model B was greater than that of Model A. The stiffness of Model B during left and right rotation was 32.8% and 36.2% higher, respectively, than that of Model A (Model B: 7.7 and 7.9 N·m/°, respectively; Model A: 5.8 and 5.8 N·m/°, respectively) (Fig. 3). The stiffness of Model B was also higher than that of Model A for left bending (5.7%), right bending (5.8%), and extension (4%).

### von Mises stress on rods and artificial pedicle fixation screws

**Fig. 4:**
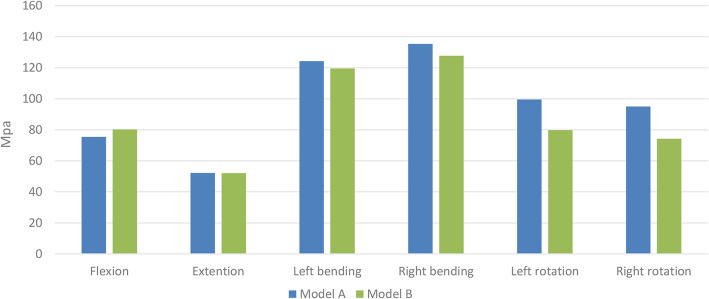
Maximum von Mises stress on posterior rods

The von Mises stress in the hardware was concentrated at the posterior rods in the two fixation models, with the maximum stress occurring during lateral bending, followed by axial rotation. In Model B, the stress on the rods decreased in all types of motion except for a slight increase (6.4%) during flexion. The artificial pedicle fixation screws significantly reduced the von Mises stress in the posterior rods during rotation (left, 24.8% and right, 28.1%) (Fig. 4). During lateral bending, the stress on the rods was slightly lower in Model B than in Model A (left, 4.0% and right, 5.9%), and reduced by 7.1% during extension.

**Fig. 5:**
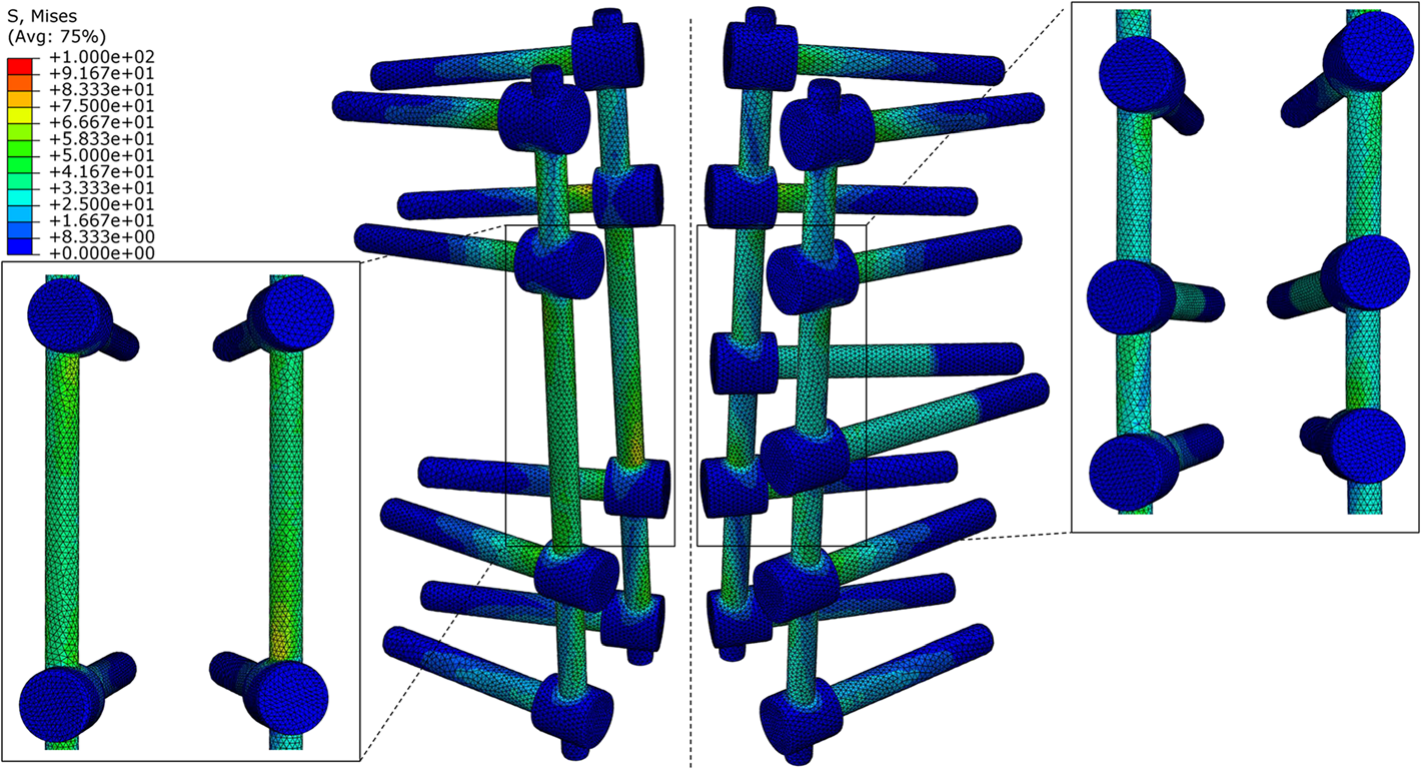
von Mises stress distribution on the posterior rods and artificial pedicle fixation screws during left rotation. Model A (left). Model B (right)

**Fig. 6:**
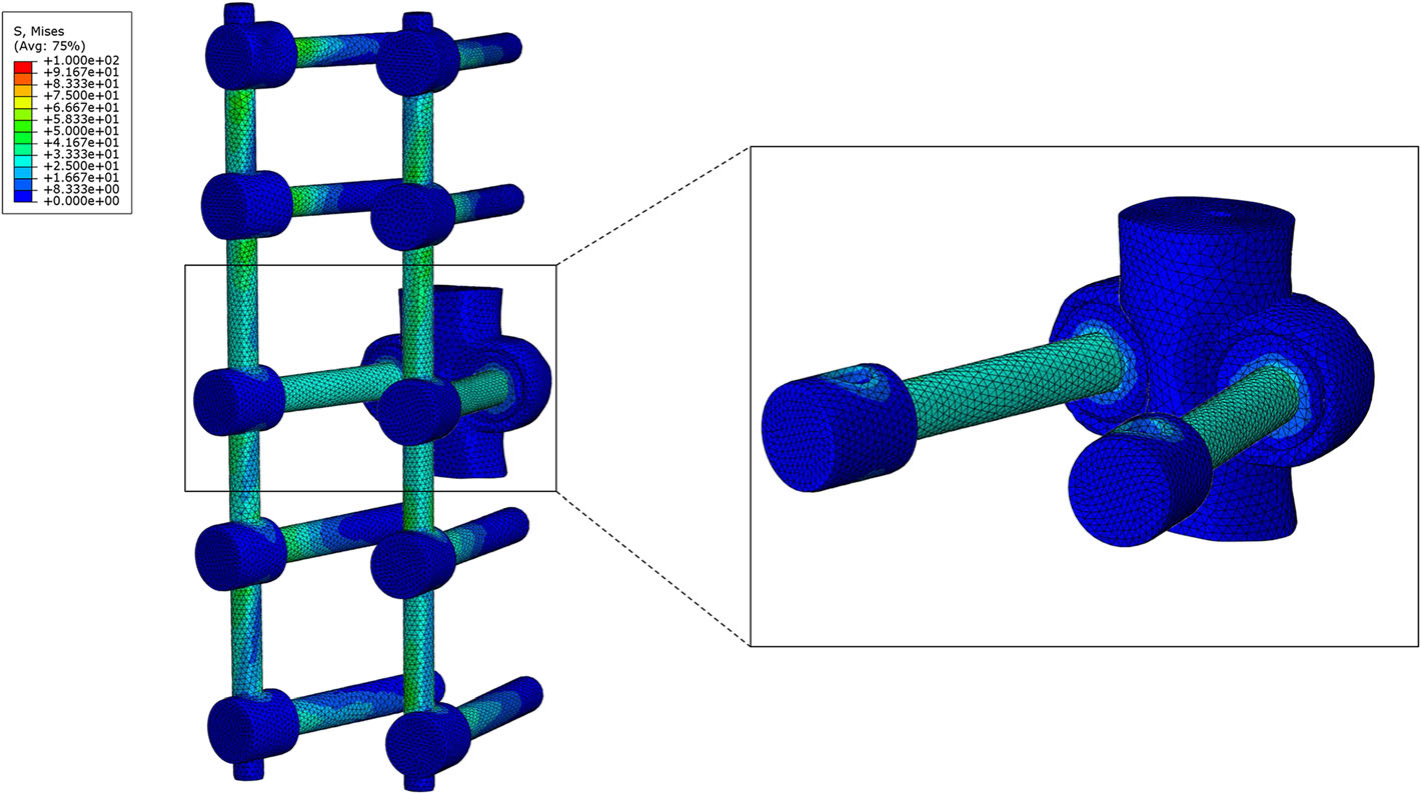
von Mises stress on the connection between the artificial pedicle of the 3D-printed prosthesis and the screws during left rotation in Model B

The artificial pedicle fixation screws of Model B had the largest von Mises stress during rotation (44.7 MPa); the stress during flexion (23.7 MPa) and bending (left 23.8 MPa, right 22.6 MPa) was only about half of that during rotation. The screws bore the least stress during extension (6.6 MPa). The artificial pedicle fixation screws more uniformly distributed the stress on the rod. The maximum stress values for Model A were 66.6 MPa at the T11/T12 segment and 87.1 MPa at the T12/L1 segment,which are located at the connection between the rods with T11 and L1 pedicle screws respectively. In contrast, in Model B, the maximum stress at T11/T12 and T12/L1 segments was 54.0 and 63.8 MPa, respectively (Fig. 5), the location of maximum stress was not changed. The maximum stress at the connection between the artificial pedicle of the 3D-printed prosthesis and the screw occurs during rotating (27.05 Mpa) (Fig. 6), and the least stress during extension(1.8Mpa). The same trends were observed for right rotation.

### von Mises stress in the endplate adjacent to the 3D-printed prosthesis

**Fig. 7:**
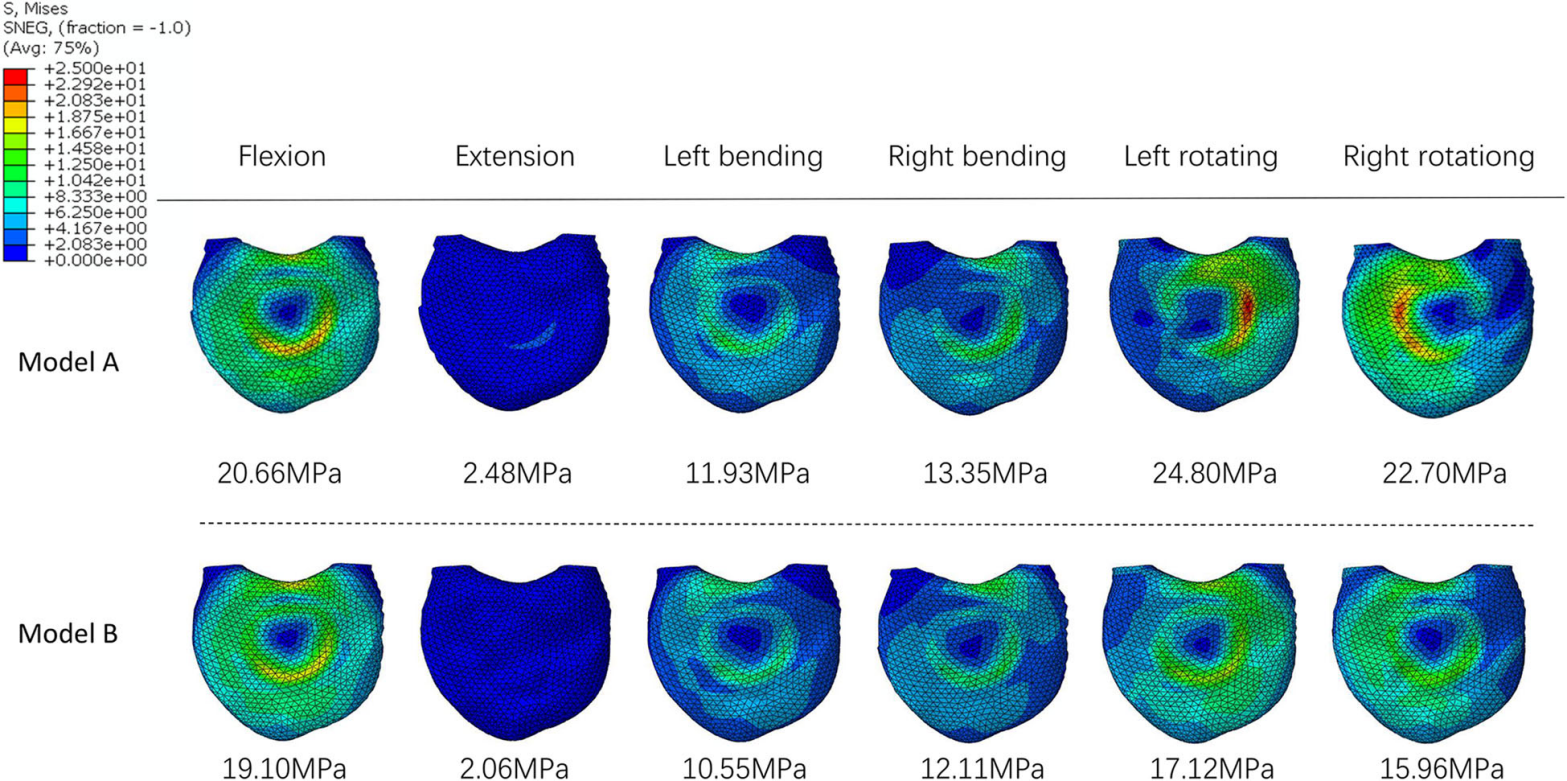
von Mises stress in the L1 superior endplate of the two fixation models

Figure 7 shows the magnitude and distribution of von Mises stress on the L1 superior endplate in the two fixation models. In Model A, the maximum stress on the endplate was during rotation. In Model B, the maximum stress was during lateral bending. Application of the artificial pedicle screws decreased the stress on the endplate during all types of motion. The most significant decrease was obtained during rotation (left, 44.8% and right, 42.2%), followed by during extension (20.4%). While there was a slight decrease in lateral bending (left, 13.1% and right, 10.2%) and flexion (8.2%).

## Discussion

Stable reconstruction of segmental defects after TES is a clinical challenge. The most commonly used anterior VBR is titanium mesh and expandable cage; its stability depends on axial pressure as it has no fixed connection to the posterior rods. A 3D-printed prosthesis can be designed and manufactured with a bionic artificial pedicle that connects to the posterior rods by screws, which would improve the stability of the fixation system. In this study, we analyzed the stability and biomechanics of the artificial pedicle structure in a 3D-printed prosthesis for stable reconstruction after T12 TES using 3D finite element models of the thoracolumbar segment (T10–L2) that simulated two different fixation methods. Fixation with the artificial pedicle enhanced the stability of the construct and reduced the stress on the posterior rods and endplate adjacent to the prosthesis. For the role of artificial pedicles fixation in the fixation system, we consider the following possibilities: (1) the addition of artificial pedicle fixation enhanced the frame structure of the anterior and psterior fixtures, which is beneficial to improve the structural stability; (2) Artificial pedicle fixation makes the anterior and posterior internal fixation fixed connection, which can conduct direct stress transmission, and reduce the stress concentration of the hardware.

In our analysis, we used construct stiffness to represent construct stability. The construct stiffness was highest during flexion followed by lateral bending and was lowest during rotation and extension. This is consistent with previous results from biomechanical tests [21]. The comparison of the two different fixation models showed that the stiffness of Model B was up to 36.2% higher than that of Model A during rotation, with a similar trend observed for other types of motion. TES causes severe spinal instability and requires rigid reconstruction to ensure later bone fusion [22]. Biomechanical testing has revealed that combining short-segment posterior fixation (with one-level pedicle screws fixation above and below the VBR) with VBR cannot stabilize the spine segment; long-segment posterior fixation provided better stability than the intact spine [6, 26]. but in long-term follow-ups, this fixation method still had a relatively high rate of hardware-associated complications, demonstrating that fixation strength must be further improved to prevent instrumentation failure [22]. In an experiment of short-segment posterior fixation after TES, connecting the anterior VBR and posterior rods with screws increased the stiffness of the fixation system by 40% [10]. Some investigators have suggested adding 3-mm threaded rods between the titanium mesh and posterior rods as artificial pedicles to enhance construct stiffness [11], and the use of a carbon fiber prosthesis that can be connected to posterior rods significantly reduced the instrumentation failure rate [27, 28]. Thus, artificial pedicle fixation has a positive effect on the stability of the fixation system. One study reported that there was no difference between the carbon fiber prosthesis and expandable cage in terms of biomechanics [21], but it failed to take into account the difference in stability of fixation for the two VBRs and adjacent endplates [29]. In our study, we evaluated long-segment posterior fixation using the same prosthesis and stress conditions and found that artificial pedicle fixation increased the stiffness of the fixation system during rotation by more than 30% by providing greater structural stability.

The main complication after TES is instrumentation failure associated with fracture of the posterior rod. In some case studies, the rod fracture rate is as high as 40% [22, 30, 31]; it has been suggested that in order to reduce anterior nonfusion and pseudoarthrosis, the construct stiffness must be increased, for instance by using thicker rods. Our results demonstrate that artificial pedicle fixation significantly alleviated stress on the posterior rods, especially during rotation, with peak stress reduced by up to 28.1%. The rod usually fractures behind the corresponding VBR [22], especially after VBR subsidence. We found that the artificial pedicle fixation screws not only increased construct stiffness, but also enabled direct stress transmission between the VBR and rod. The stress of the artificial pedicle screw during rotation (44.7 MPa) was more than twice that during flexion (23.7 MPa) and lateral bending (22.6 MPa), which significantly diminished the stress on the posterior rods during rotation and redistributed the stress more evenly across these rods (Fig. 5). The position of the maximum stress on the rod of T11–L1 segment has not changed, which is higher than the connection between the artificial pedicle fixed screw with the rod and the prosthesis. Therefore, artificial pedicle screw fixation did not cause stress concentration. Because of the overall stress reduction, the fixed system was more stable and less prone to metal fatigue under long-term load, which can extend service life. Enhancing the stability of the fixation system by applying artificial pedicle fixation and thereby reducing the stress on the posterior rods is more conducive to anterior fusion and decreases the risk of internal fixation failure.

Higher interfacial stress is an important factor contributing to cage subsidence [32, 33]. VBR subsidence is the major complication after TES (accounting for 63% of all complications) [34] and can lead to instrumentation failure [8, 9]. Better results have been achieved by increasing the surface area of the VBR and enlarging the contact area with the endplate to alleviate stress concentration [35, 36]. In addition to increasing control of the VBR and enhancing system stability, short-segment posterior fixation with an artificial pedicle was shown to reduce the subsidence of cancellous bone by 50% [10]. In our experiment, artificial pedicle fixation reduced the stress of adjacent endplates by up to 44.8% during rotation. The stability of the VBR without artificial pedicle fixation mainly depends on the compression force of the screws and the axial gravity of the body. As the rotational force is perpendicular to the stability force of the VBR, so the VBR and the endplate need to maintain a larger contact stress. The artificial pedicle fixation screws enabled transmission of stress between the rod and VBR (Fig. 4), which greatly reduced the stress on the VBR–endplate. Even with a customized 3D prosthesis, it may be difficult to achieve a perfect fit between the prosthesis and endplate because the former may change position during placement. Adding artificial pedicle fixation is a convenient and effective way to increase fixation and reduce endplate stress and prosthesis subsidence.

There were some limitations to our study. Our experiment involved finite element analysis, which has certain shortcomings. Firstly, the models assumed the structure of the vertebral body to be homogeneous and isotropic, and finite element modeling data obtained from a single image may not reflect individual differences in a population. Secondly, our research had only one object in each model, according to the research by Li et al. [37] and Liang et al. [38], a difference more than 20% of differences was considered “important” or “relevant,” so we define differences over 20% as “significant.” Additionally, the connection between the prosthesis and bone interface ignores any possible displacement. Finally, the finite element model did not include muscles and surrounding soft tissues and therefore did not allow an accurate analysis of spine forces. Further biomechanical testing using biological specimens is needed to validate our findings.

## Conclusion

After thoracolumbar TES, the two long-segment posterior fixation combined with the anterior VBR methods could maintain the postoperative spinal stability. The application of artificial pedicle fixation in the 3D printed prosthesis increased the stiffness of the fixation system and reduced the stress on the posterior rods and endplate adjacent to the 3D-printed prosthesis. These results support that the use of artificial pedicle fixation improves the stability of the spinal fixation system and reduces the risk of prosthesis subsidence and instrumentation failure.

## Data Availability

The datasets used and/or analyzed during the current study are available from the corresponding author on reasonable request.

## Abbreviations

TES: Total en bloc spondylectomy
VBR: Vertebral body replacement
3D: 3-dimensional
DICOM: Digital Imaging and Communications in Medicine
CT: Computed tomography
IVD: Intervertebral disc
ROM: Range of motion
